# Supplementary material for: A herpesvirus encoded Qa-1 mimic inhibits natural killer cell cytotoxicity through CD94/NKG2A receptor engagement
Source: eLife. 2018 Dec 21;7:e38667. doi: 10.7554/eLife.38667 (PMC6320069; doi:10.7554/eLife.38667)
Supplement: Supplementary file 1. [file elife-38667-supp1.docx]

**Supplementary File 2. Peptides used for pQa-1 surface stabilization assay**

| **Peptide** | **Position** | **Protein [organism]** |
| --- | --- | --- |
| AMAPRTLLL (Qdm) | 3-11 3-11 3-11 3-11 3-11 44-52 7-15 | H-2 D, L [Mus musculus], [Mus caroli]  RT1 class I [Rattus norvegicus] H-2 Q10 like [Microtus ochrogaster]  HLA-C [Pongo abelii]  MHC class I antigen [Pongo pygmaeus], [Macaca leonina] H-2 Q10 like [Meriones unguiculatus]  Hemagglutinin-esterase [Murine and rat coronavirus , murine hepatitis virus] |
| GMAPRTLLL | 3-11 3-11 | MHC class I [macaca nemestrina] BOLA class I histocompatibility antigen [Physeter catodon] |
| SMAPRTLLL | 3-11 3-11 3-11 | H-2 Q10 [Microtus ochrogaster] HLA-B [Ochotona princeps] RLA class I 19-1 [Oryctolagus cuniculus] |
| VMAPRTLLL | 3-11 3-11 3-11 53-61 55-63 8-16 8-16 | MHC-B [Papio Anubis] HLA-A [Homo sapiens] MHC class I [Macaca mulatta] class I histocompatibility antigen [Papio anubis] HLA-G [Pongo abelii]  Membrane glycoprotein UL40 [Cercopithecine herpesvirus 5] rh67 [Macacine herpesvirus 3] |
| MAPRTLLL | 1-8 | Predicted manu MHC class I antigen |
| AVAPRTLLL | 3-11 3-11 115-123 138-146 | MHC class I antigen [Cricetulus griseus] H-2 Q10 like, L-D like [Microtus ochrogaster]  H2-L [Cricetulus griseus]  H2-T23 [Cricetulus griseus] |
| AMVPRTLLL (Qdm-k) | 3-11 | H-2 Dk [Mus musculus] |
