# Supplementary material for: A herpesvirus encoded Qa-1 mimic inhibits natural killer cell cytotoxicity through CD94/NKG2A receptor engagement
Source: eLife. 2018 Dec 21;7:e38667. doi: 10.7554/eLife.38667 (PMC6320069; doi:10.7554/eLife.38667)
Supplement: Supplementary file 2. [file elife-38667-supp2.docx]

**Supplementary File 1. RHVP encodes a protein with high identity to Qa-1***


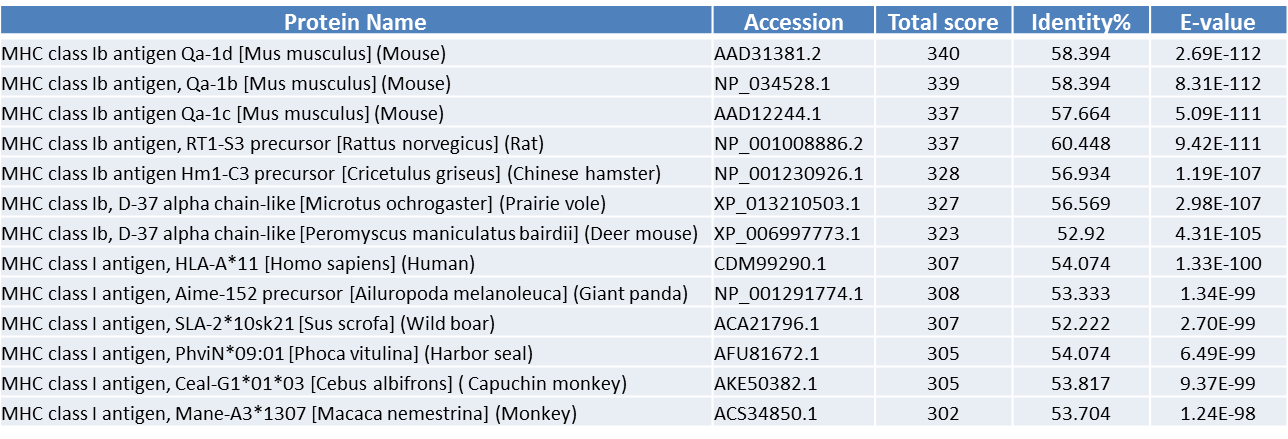


*The putative protein of RHVP encoded by 1383-236 nucleotides in reverse direction was searched against non-redundant protein sequences using NCBI/BLAST server. The top 100 hits after deletion of isoforms or allelic variations are ranked by total score.
